# Supplementary material for: Chlorophyll Derivatives from Marine Cyanobacteria with Lipid-Reducing Activities
Source: Mar Drugs. 2019 Apr 17;17(4):229. doi: 10.3390/md17040229 (PMC6520785; doi:10.3390/md17040229)
Supplement: Supplementary file 1 [file marinedrugs-17-00229-s001.pdf]

Supplementary Materials

Chlorophyll derivatives from marine cyanobacteria with lipid reducing activities

Sara Freitas [1,2,+,*], Natália Gonçalves Silva [1,+,*], Maria Lígia Sousa [1], Tiago Ribeiro [1], Filipa Rosa [1], Pedro N. Leão [1], Vitor Vasconcelos [1,2], Mariana Alves Reis [1] and Ralph Urbatzka [1,2,*]

[1] Interdisciplinary Center of Marine and Environmental Research (CIIMAR/CIMAR), University of Porto, Terminal de Cruzeiros de Leixões, Av. General Norton de Matos s/n, 4450-208 Matosinhos, Portugal; sfreitas@ciimar.up.pt (S.F.), nsilva@ciimar.up.pt (N.G.S.), msousa@ciimar.up.pt (M.L.S.), tribeiro@ciimar.up.pt (T.R.), frosa@ciimar.up.pt (F.R.), pleao@ciimar.up.pt (P.N.L.), vmvascon@fc.up.pt (V.V.), mareis@ciimar.up.pt.com (M.R.), rurbatzka@ciimar.up.pt (R.U.)

[2] FCUP, Faculty of Science, Department of Biology, University of Porto, Rua do Campo Alegre, 4169-007, Porto, Portugal;

[+] Joint first authors: sfreitas@ciimar.up.pt (S.F.); nsilva@ciimar.up.pt (N.G.S.);

* Correspondence: rurbatzka@ciimar.up.pt; Tel.: +351 223 401 818

Received: date; Accepted: date; Published: date

List of Contents

Figure S1. ^{1}H NMR spectrum of 13^2-hydroxy-pheophytin a, compound **1**, in DMSO-*d6* (400 MHz).

Figure S2. ^{13}C NMR spectrum of 13^2-hydroxy-pheophytin a, compound **1**, in DMSO-*d6* (400 MHz).

Figure S3. HSQC spectrum of 13^2-hydroxy-pheophytin a, compound **1**, in DMSO-*d6* (400 MHz).

Figure S4. HMBC spectrum of 13^2-hydroxy-pheophytin a, compound **1**, in DMSO-*d6* (400 MHz).

Figure S5. ^{1}H-^{1}H COSY spectrum of 13^2-hydroxy-pheophytin a, compound **1**, in DMSO-*d6* (400 MHz).

Figure S6. Full LC-ESI-HRMS spectrum of 13^2-hydroxy-pheophytin a, compound **1**, in the positive mode.

Figure S7. LC-ESI-HRMS/MS spectrum of 13^2-hydroxy-pheophytin a, compound **1**, in the positive mode, showing the major fragments *m/z* 869.5542 [M – OH] $^+$, *m/z* 609.2696 [M – phytol] $^+$, *m/z* 591.2602 [M – OH-phytol]$^+$.

Figure S8. ^{1}H NMR spectrum of 13^2-hydroxy-pheofarnesin a, compound **2**, in $CDCl_3$ (600 MHz).

Figure S9. ^{13}C NMR spectrum of 13^2-hydroxy-pheofarnesin a, compound **2**, in $CDCl_3$ (600 MHz).

Figure S10. HSQC spectrum of 13^2-hydroxy-pheofarnesin a, compound **2**, in $CDCl_3$ (600 MHz).

Figure S11. HMBC spectrum of 13^2-hydroxy-pheofarnesin a, compound **2**, in $CDCl_3$ (600 MHz).

Figure S12. ^{1}H-^{1}H COSY spectrum of 13^2-hydroxy-pheofarnesin a, compound **2**, in $CDCl_3$ (600 MHz).

Figure S13. ROESY spectrum 13^2-hydroxy-pheofarnesin a, compound **2**, in DMSO-*d6* (600 MHz).

Figure S14. Full LC-ESI-HRMS spectrum of 13^2-hydroxy-pheofarnesin a, compound **2**, in the positive mode.

Figure S15. LC-ESI-HRMS chromatogram of 13^2-hydroxy-pheofarnesin a, compound **2**, in the positive mode, showing major fragments *m/z* 840.4339 [M + H + Na]$^+$, 609.2697 [M +2H – farnesyl]$^+$, 591.2604 [M – OH – farnesyl]$^+$.

Figure S16. 3T3-L1 organoids without differentiation induction (upper images) and after differentiation induction (lower images). Differentiated organoids were formed during 5 days in DMEM medium and then exposed for 3 days to a differentiated medium, containing 10 µg/ml of insulin, 250 nM dexamethasone and 500 µM of isobutylmethylxanthine. After this step, an exposure assay to 1 or 2 can take place. Not differentiation organoids were cultured as differentiated organoids but without the differentiation medium.

Figure S17. LC-ESI-HRMS/MS chromatogram comparing compound **1** in 5 different alga- plant-based materials, as well as standard compound. Samples were prepared at 0.2 mg/ml in MeOH (100%). Presence of the major fragments of **1** in all samples confirm its presence in the alga- plant-based materials (HR-ESI-MS/MS *m/z* 869.5542 [M – OH] +, *m/z* 609.2696 [M – phytol] +, *m/z* 591.2602 [M – OH-phytol]+).

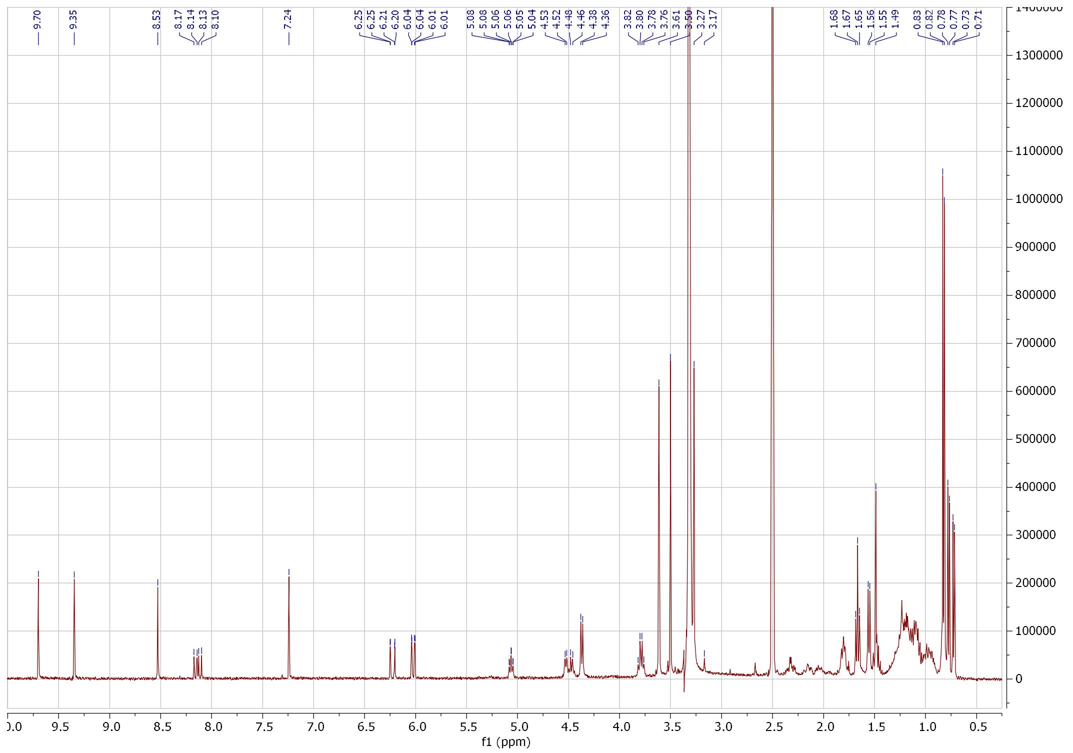

Figure S 1 - ^{1}H NMR spectrum of 13^2-hydroxy-pheophytin a, compound **1**, in DMSO-*d6* (400 MHz).

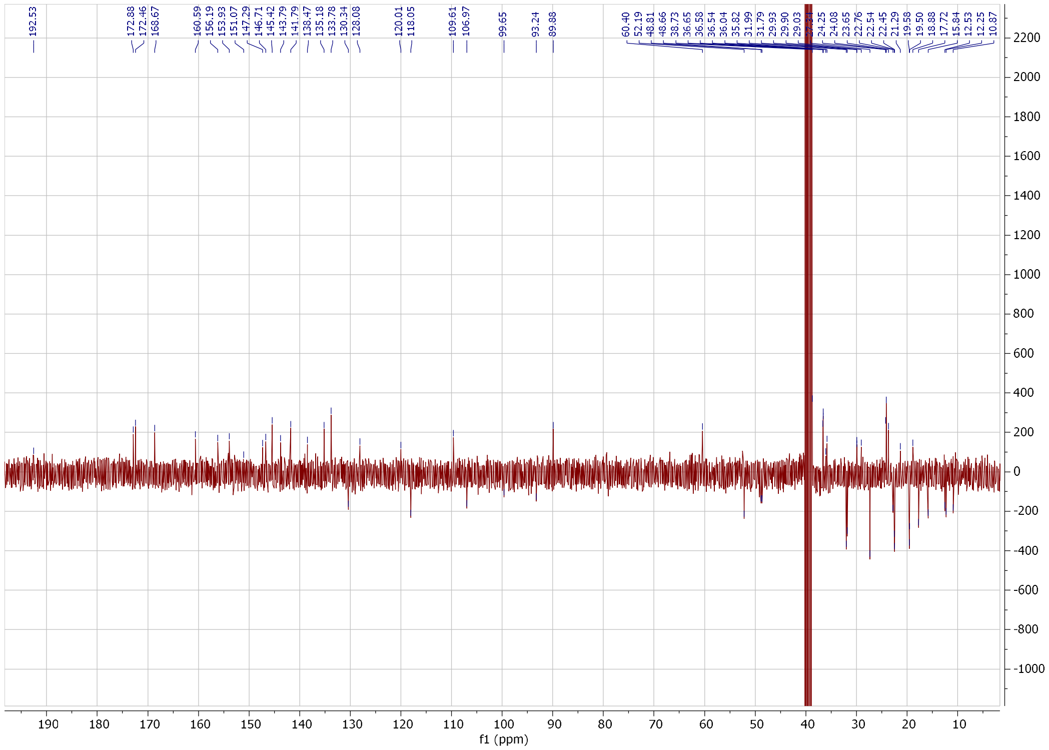

Figure S 2 - ^{13}C NMR spectrum of 13[2]-hydroxy-pheophytin a, compound **1**, in DMSO-*d6* (400 MHz).

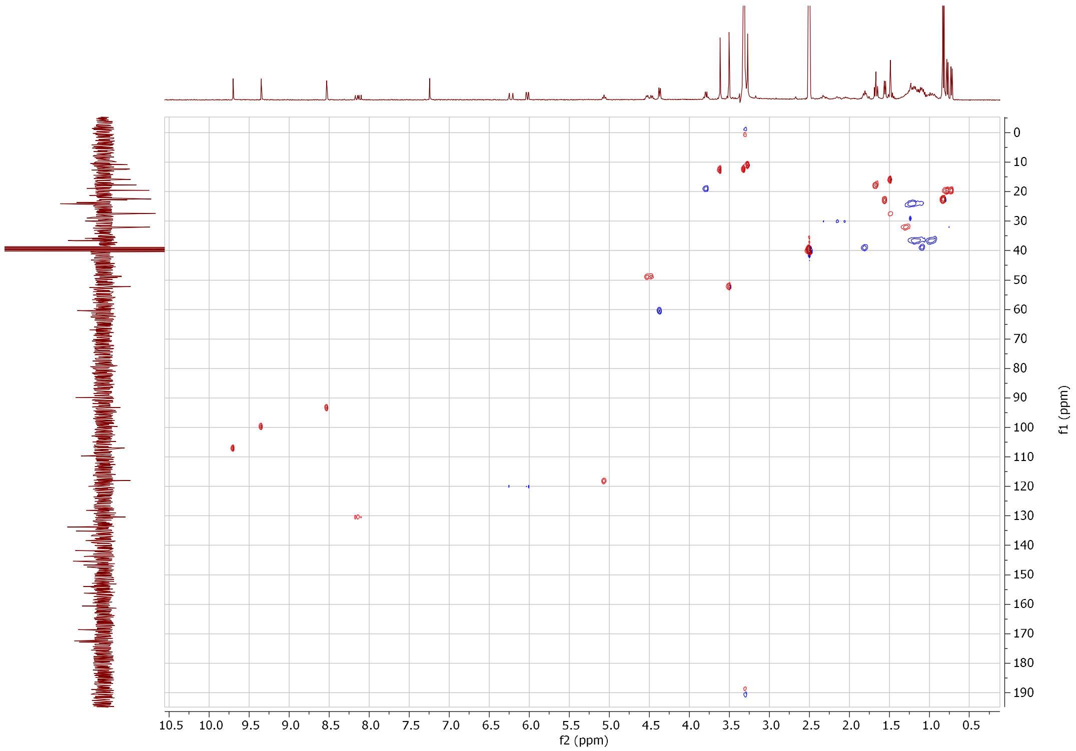

Figure S 3 - HSQC spectrum of 13^2-hydroxy-pheophytin a, compound **1**, in DMSO-d6 (400 MHz).

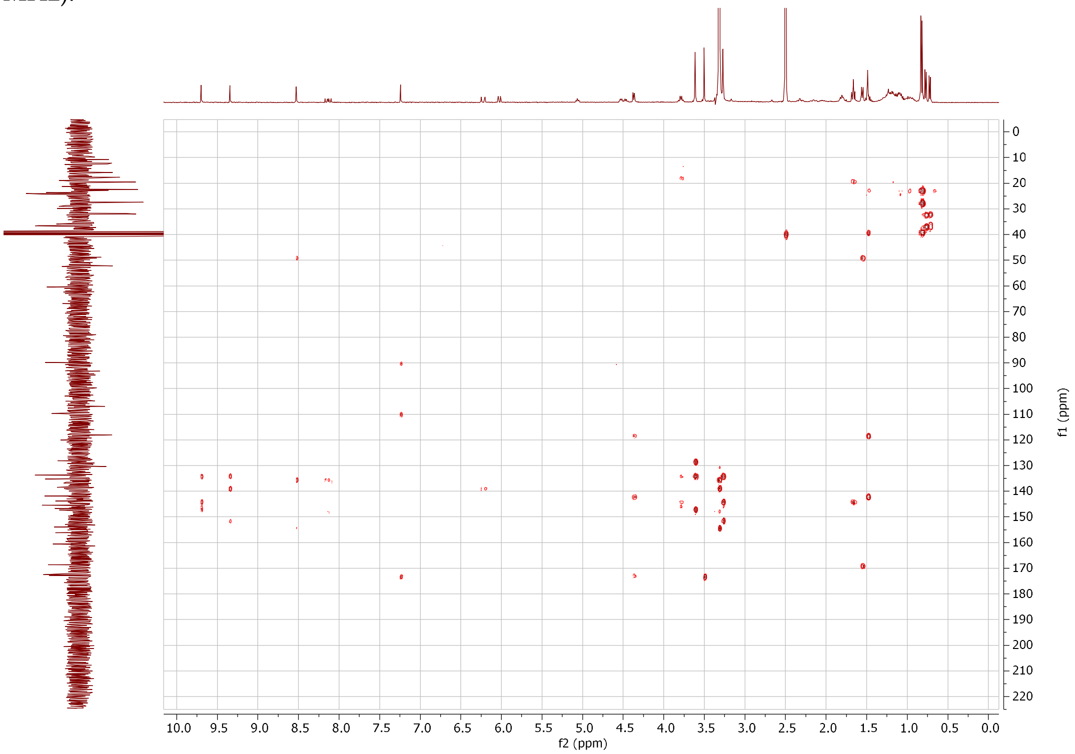

Figure S 4 - HMBC spectrum of 13^2-hydroxy-pheophytin a, compound **1**, in DMSO-*d*6 (400 MHz).

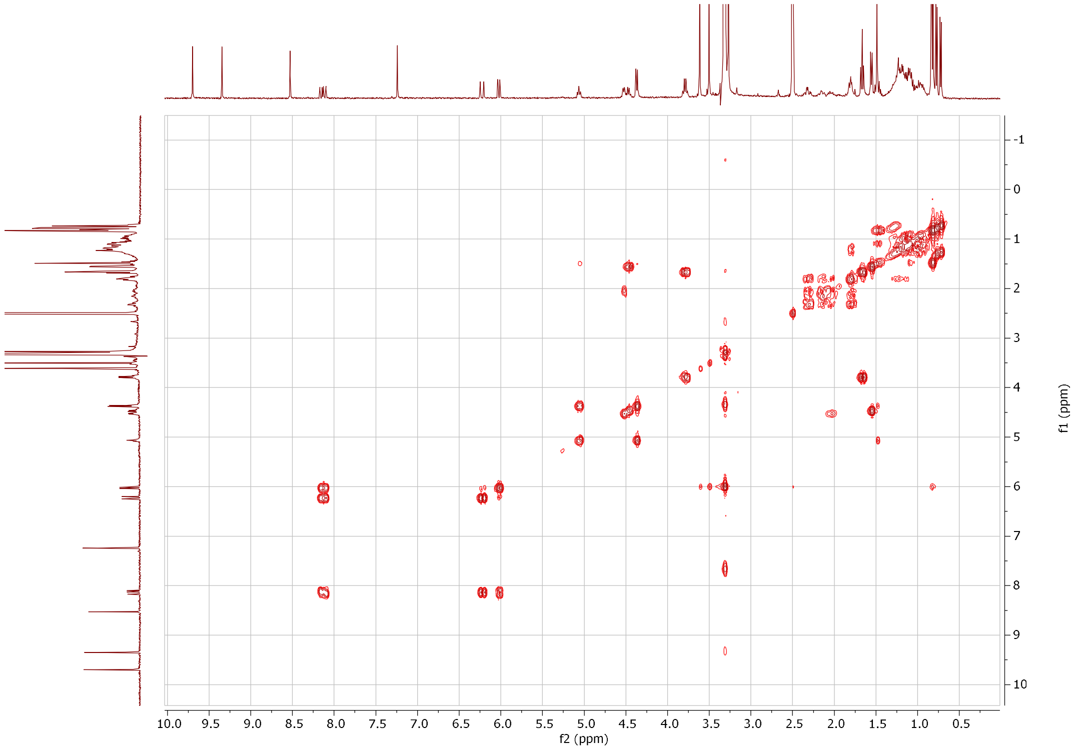

Figure S 5 - ^{1}H-^{1}H COSY spectrum of 13²-hydroxy-pheophytin a, compound **1**, in DMSO-*d6* (400 MHz).

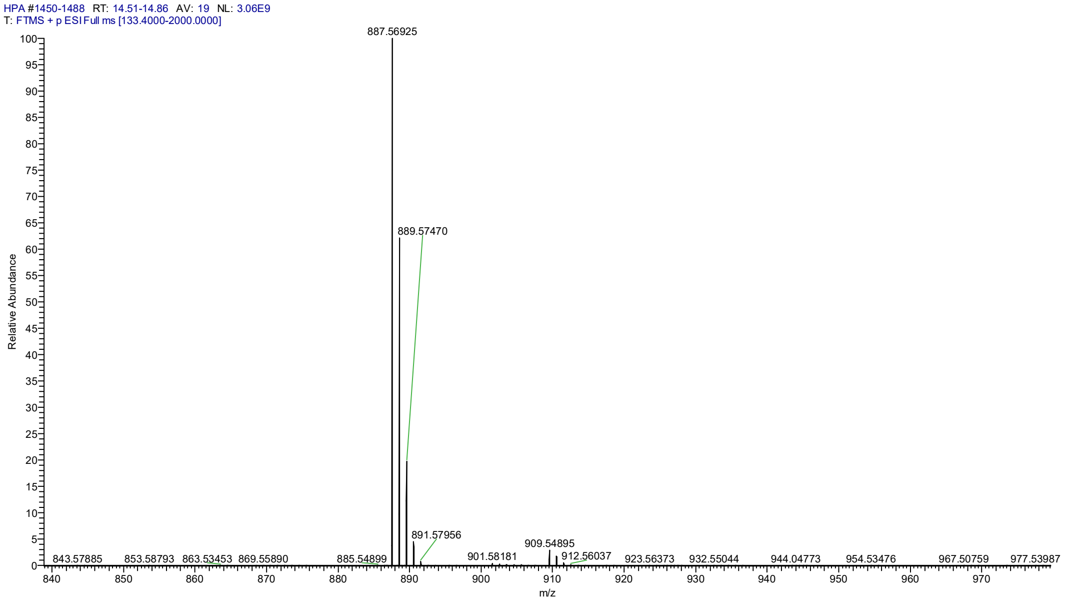

Figure S 6 - Full LC-ESI-HRMS spectrum of 13²-hydroxy-pheophytin a, compound **1**, in the positive mode.

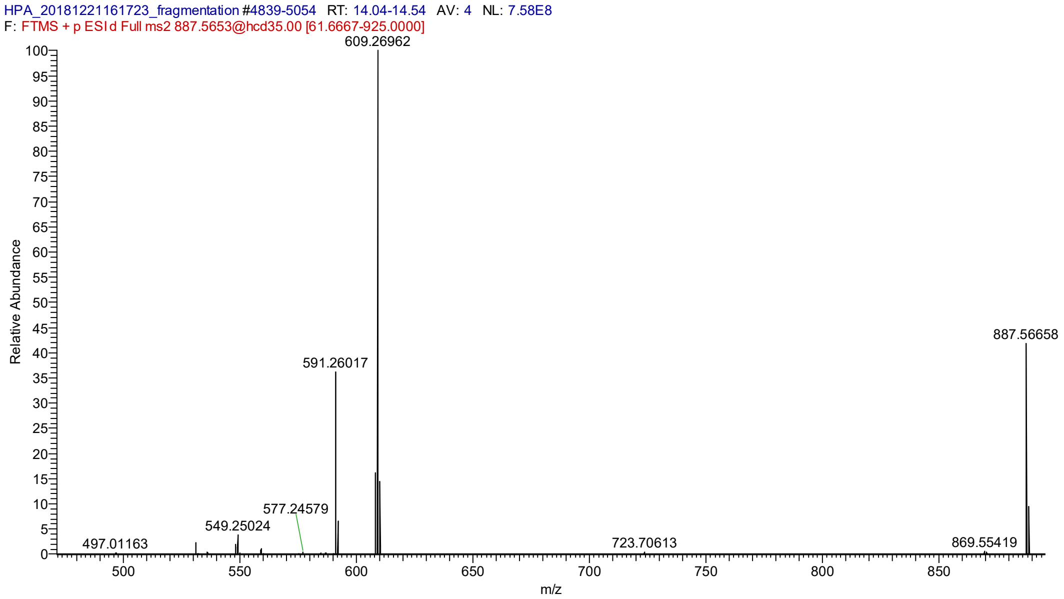

Figure S 7 - LC-ESI-HRMS/MS spectrum of 13^2-hydroxy-pheophytin a, compound **1**, in the positive mode, showing the major fragments *m/z* 869.5542 [M – OH] $^+$, *m/z* 609.2696 [M – phytol] $^+$, *m/z* 591.2602 [M – OH – phytol]$^+$.

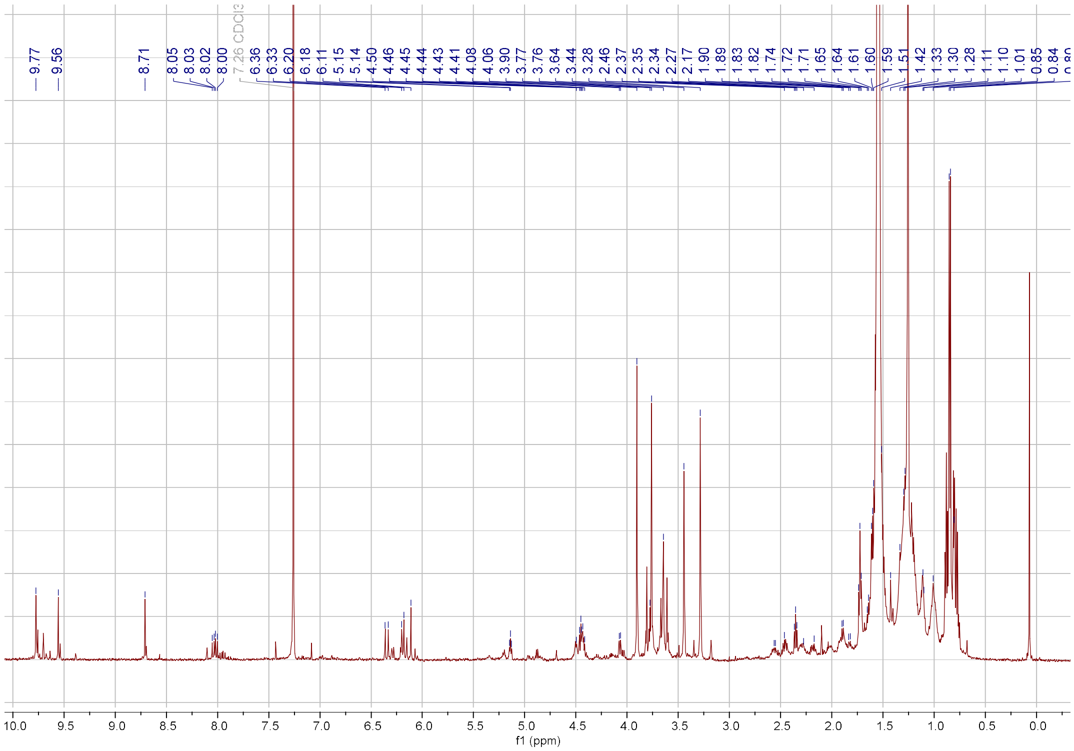

Figure S 8 - ^{1}H NMR spectrum of 13^2-hydroxy-pheofarnesin a, compound **2**, in $CDCl_3$ (600 MHz).

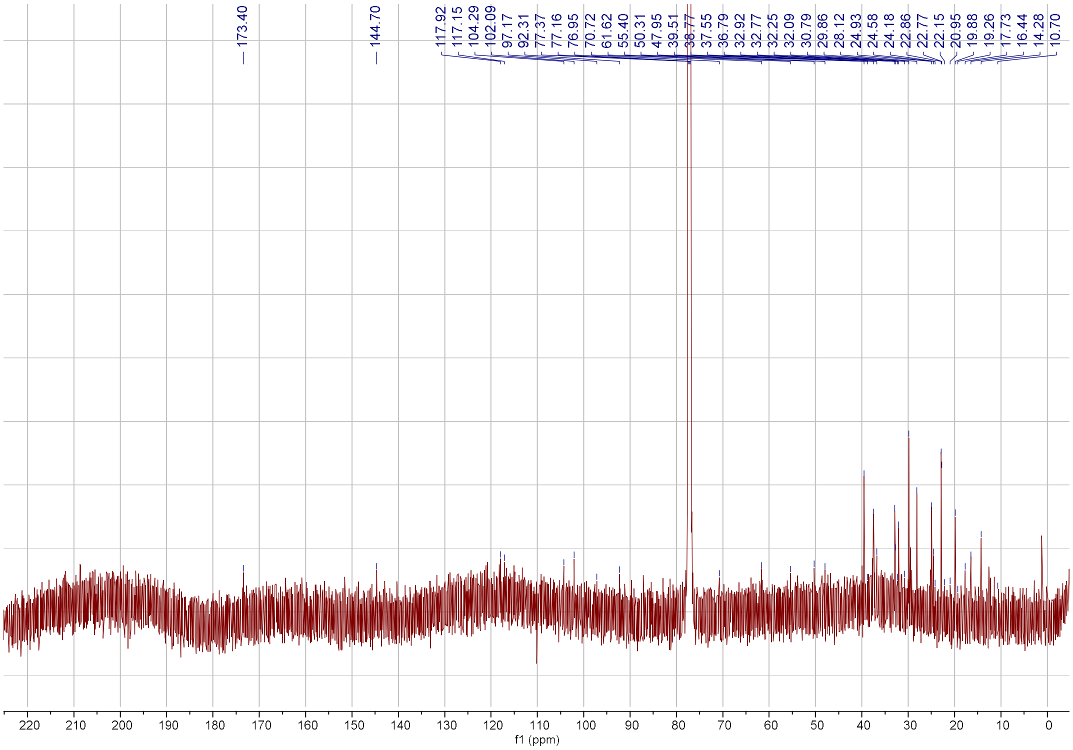

Figure S 9 - ^{13}C NMR spectrum of 13^2-hydroxy-pheofarnesin a, compound **2**, in $CDCl_3$ (600 MHz).

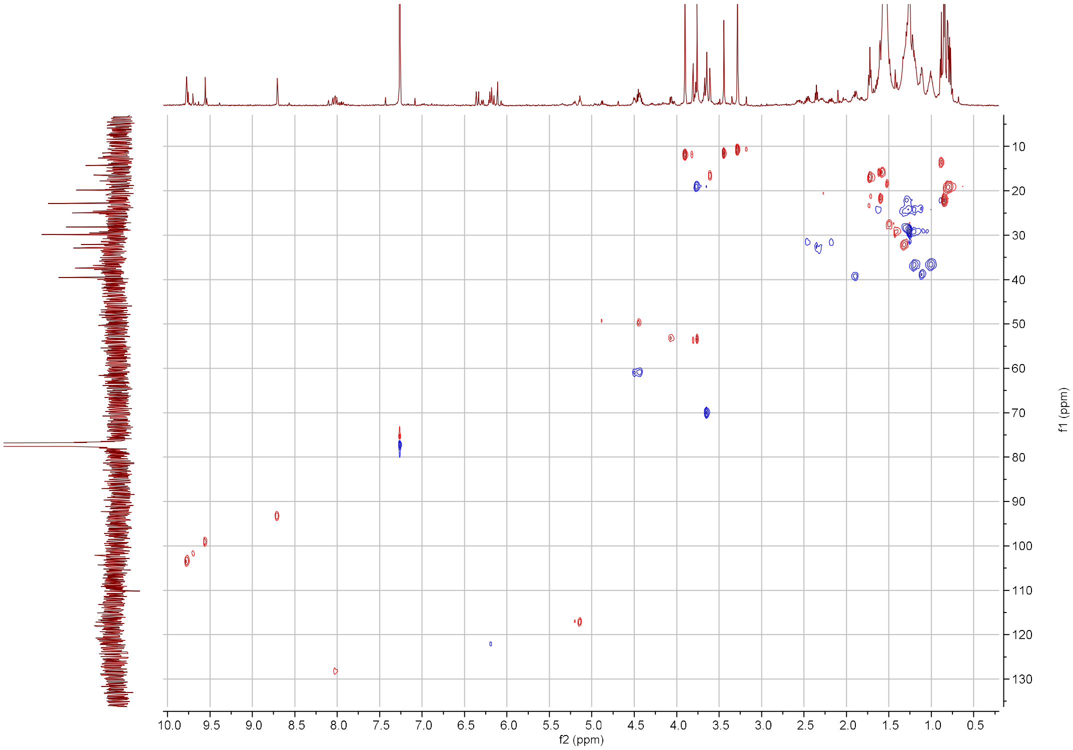

Figure S 10 - HSQC spectrum of 13^2-hydroxy-pheofarnesin a, compound **2**, in $CDCl_3$ (600 MHz).

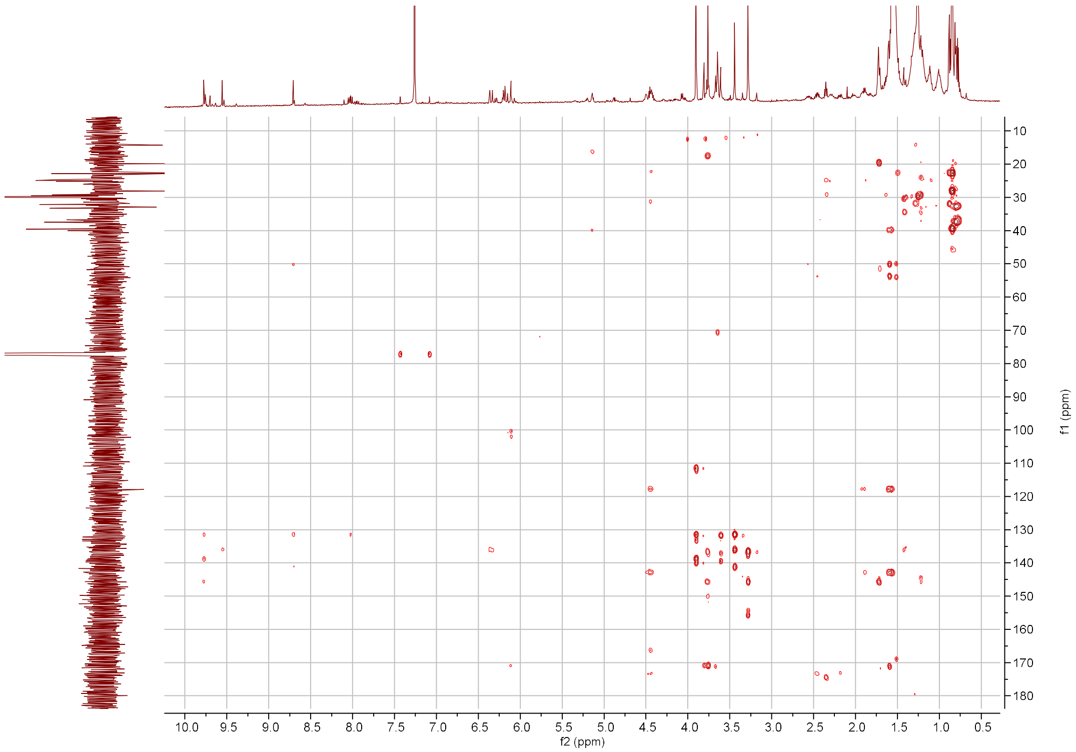

Figure S 11 - HMBC spectrum of 13²-hydroxy-pheofarnesin a, compound **2**, in $CDCl_3$ (600 MHz).

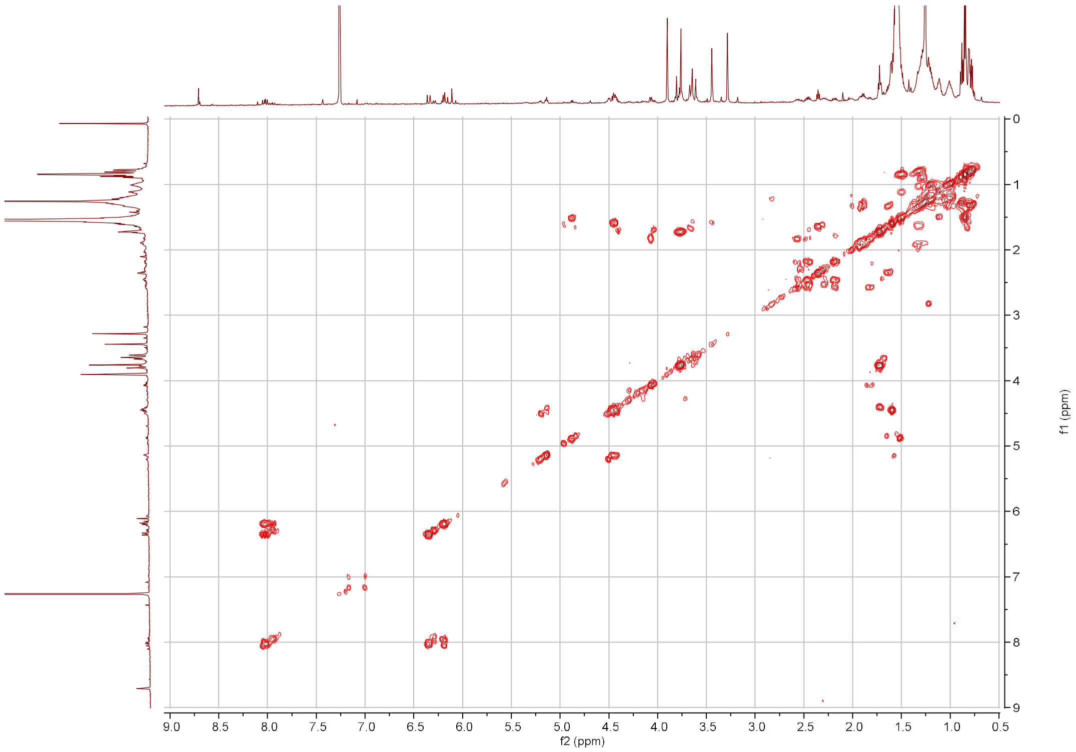

Figure S 12 - ^1H-^1H COSY spectrum of 13²-hydroxy-pheofarnesin a, compound **2**, in $CDCl_3$ (600 MHz).

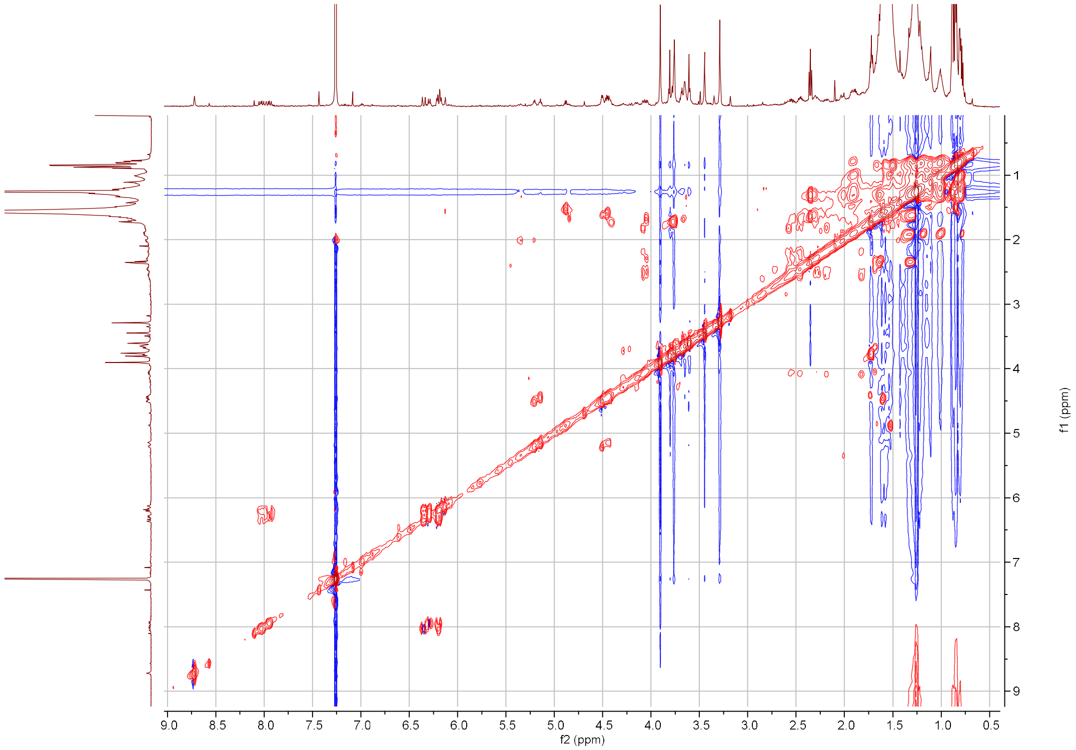

Figure S 13 - ROESY spectrum 13²-hydroxy-pheofarnesin a, compound **2**, in DMSO-*d6* (600 MHz).

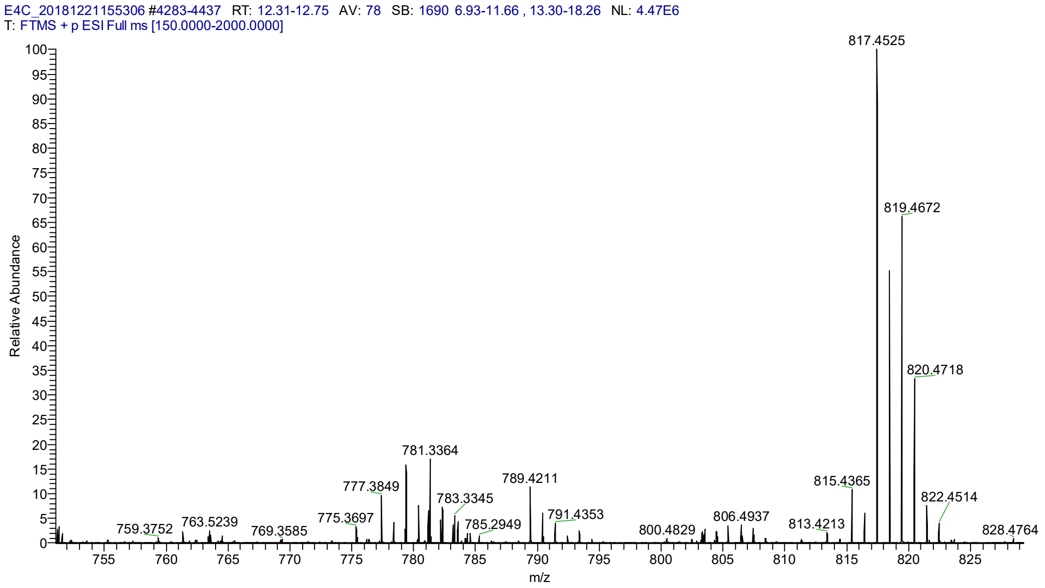

Figure S 14 - Full LC-ESI-HRMS spectrum of 13²-hydroxy-pheofarnesin a, compound **2**, in the positive mode.

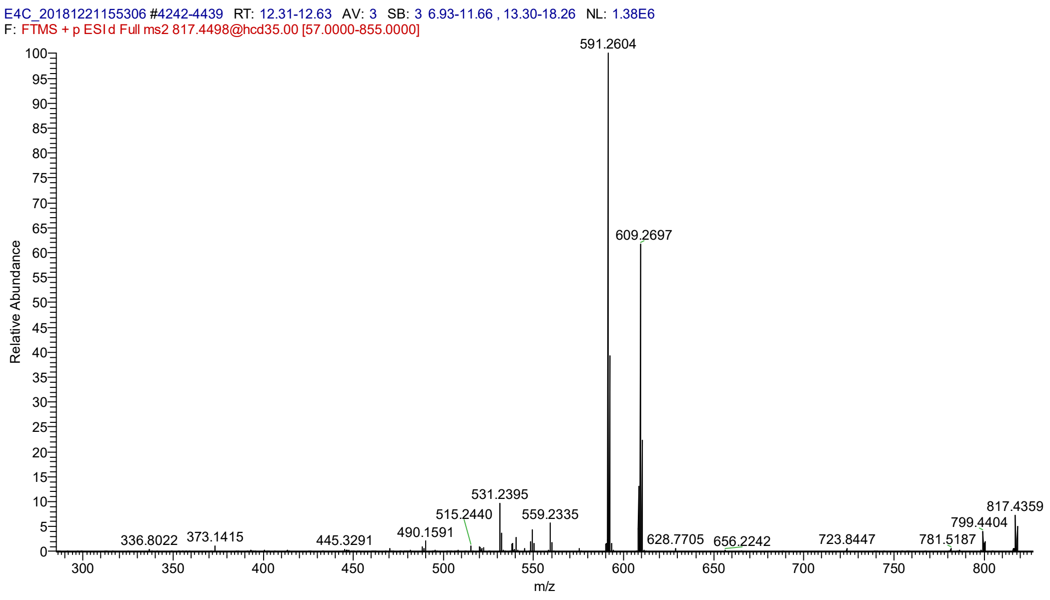

Figure S 15 - LC-ESI-HRMS chromatogram of 13^2-hydroxy-pheofarnesin a, compound **2**, in the positive mode, showing major fragments *m/z* 840.4339 $[M + H + Na]^+$, 609.2697 $[M + 2H - \text{farnesyl}]^+$, 591.2604 $[M - OH - \text{farnesyl}]^+$.

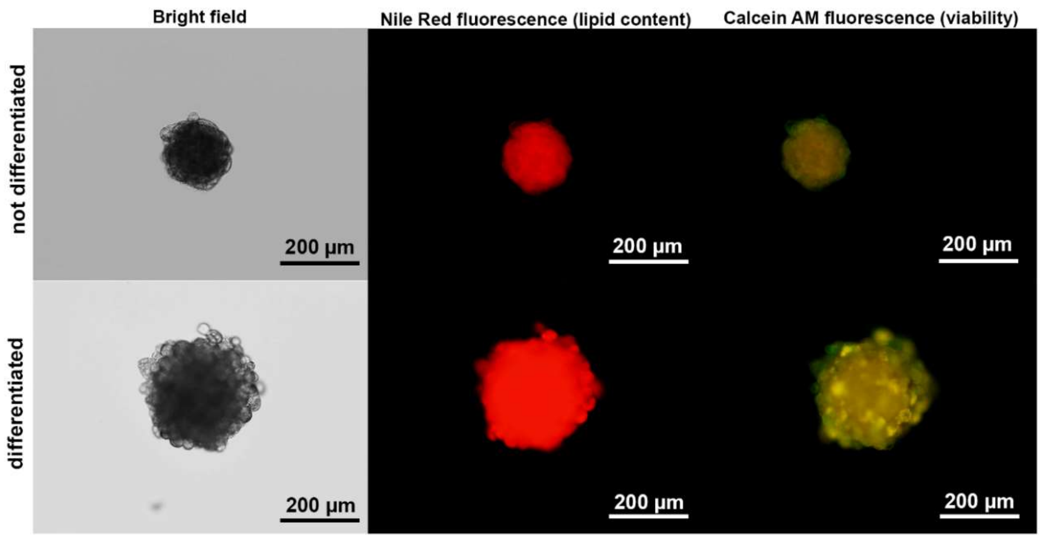

Figure S 16 - 3T3-L1 organoids without differentiation induction (upper images) and after differentiation induction (lower images). Differentiated organoids were formed during 5 days in DMEM medium and then exposed for 3 days to a differentiated medium, containing 10 µg/ml of insulin, 250 nM dexamethasone and 500 µM of isobutylmethylxanthine. After this step, an exposure assay to 1 or 2 can take place. Not differentiation organoids were cultured as differentiated organoids but without the differentiation medium.

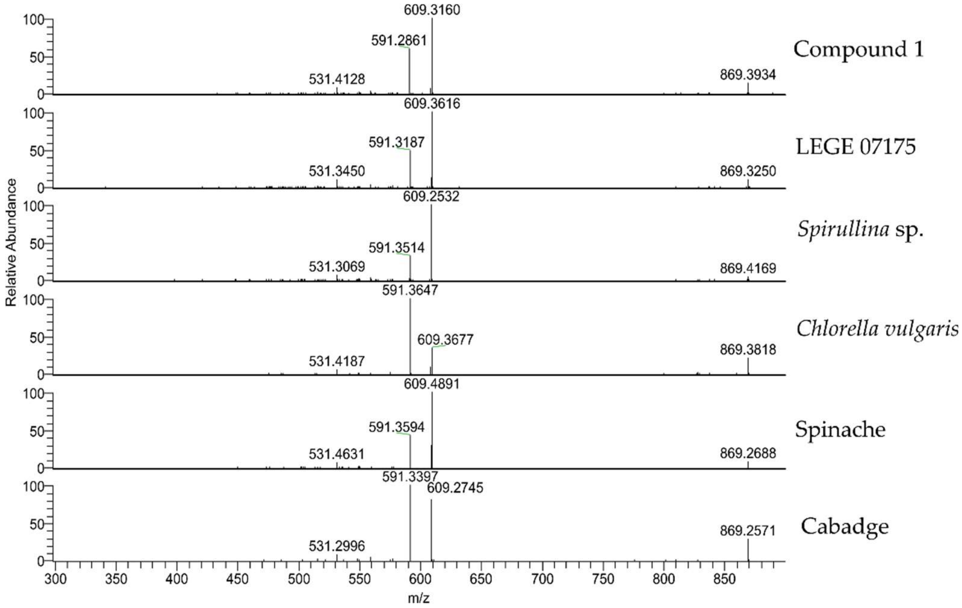

Figure S 17 – LC-ESI-HRMS/MS chromatogram comparing compound **1** in 5 different alga- plant- based materials, as well as standard compound. Samples were prepared at 0.2 mg/ml in MeOH (100%). Presence of the major fragments of **1** in all samples confirm its presence in the alga- plant- based materials (HR-ESI-MS/MS *m/z* 869.5542 $[M - OH]^+$, *m/z* 609.2696 $[M - phytol]^+$, *m/z* 591.2602 $[M - OH - phytol]^+$).
